# Personalized Adaptive Training Improves Performance at a Professional First-Person Shooter Action Videogame

**DOI:** 10.3389/fpsyg.2021.598410

**Published:** 2021-06-10

**Authors:** Francesco Neri, Carmelo Luca Smeralda, Davide Momi, Giulia Sprugnoli, Arianna Menardi, Salvatore Ferrone, Simone Rossi, Alessandro Rossi, Giorgio Di Lorenzo, Emiliano Santarnecchi

**Affiliations:** ^1^Siena Brain Investigation & Neuromodulation Lab, Department of Medicine, Surgery and Neuroscience, Neurology and Clinical Neurophysiology Section, University of Siena, Siena, Italy; ^2^Human Physiology Section, Department of Medicine, Surgery and Neuroscience, University of Siena, Siena, Italy; ^3^Department of Medicine, Surgery and Neuroscience, University of Siena, Siena, Italy; ^4^Laboratory of Psychophysiology and Cognitive Neuroscience, Chair of Psychiatry, Department of Systems Medicine, University of Rome Tor Vergata, Rome, Italy; ^5^Psychiatry and Clinical Psychology Unit, Fondazione Policlinico Tor Vergata, Rome, Italy; ^6^IRCCS Fondazione Santa Lucia, Rome, Italy; ^7^Berenson-Allen Center for Non-Invasive Brain Stimulation, Beth Israel Deaconess Medical Center, Harvard Medical School, Boston, MA, United States

**Keywords:** videogame, first-person shooter, videogame training, cognitive training, human learning

## Abstract

First-Person Shooter (FPS) game experience can be transferred to untrained cognitive functions such as attention, visual short-term memory, spatial cognition, and decision-making. However, previous studies have been using off-the-shelf FPS games based on predefined gaming settings, therefore it is not known whether such improvement of in game performance and transfer of abilities can be further improved by creating a in-game, adaptive in-game training protocol. To address this question, we compared the impact of a popular FPS-game (Counter-Strike:Global-Offensive–CS:GO) with an *ad hoc* version of the game based on a personalized, adaptive algorithm modifying the artificial intelligence of opponents as well as the overall game difficulty on the basis of individual gaming performance. Two groups of FPS-naïve healthy young participants were randomly assigned to playing one of the two game versions (11 and 10 participants, respectively) 2 h/day for 3 weeks in a controlled laboratory setting, including daily in-game performance monitoring and extensive cognitive evaluations administered before, immediately after, and 3 months after training. Participants exposed to the adaptive version of the game were found to progress significantly faster in terms of in-game performance, reaching gaming scenarios up to 2.5 times more difficult than the group exposed to standard CS:GO (*p* < 0.05). A significant increase in cognitive performance was also observed. Personalized FPS gaming can significantly speed-up the learning curve of action videogame-players, with possible future applications for expert-video-gamers and potential relevance for clinical-rehabilitative applications.

## Introduction

Videogame consoles are widely used by adolescents and children nowadays, with up to the 60% of 8–18 years old adolescents playing videogames on a daily basis, and a great increase of the overall daytime spent playing videogames from 1999 to 2020 (Lu et al., 2012; Özçetin et al., 2019). This has consequently led to an increase in research efforts addressing both potential harms and benefits of videogaming on cognitive functions (Oei and Patterson, 2013). The vast majority of previous studies has focused on action videogames, a category characterized by complex visual scenes and different time-locked goals (Bavelier et al., 2012; Latham et al., 2013). These games require fast responses to visual and auditory cues, constant selection between multiple action plans, and peripheral processing, ultimately resulting in a very demanding perceptual load (Green et al., 2010; Hubert-Wallander et al., 2011).

Previous investigations have reported expert action videogames players (AVGPs) to outperform non-players (NAVGPs) in a variety of cognitive and perceptual tasks, including visual selective attention (Green and Bavelier, 2003, 2006a; Feng et al., 2007; Dye et al., 2009; Spence and Feng, 2010; Clark et al., 2011; Belchior et al., 2013), visual stimuli detection (Vallett et al., 2013), visual search efficiency (Castel et al., 2005), contrast sensitivity (Li et al., 2009), visual interference suppression (Hazarika et al., 2018), shifting (Green et al., 2010), cognitive flexibility (Colzato et al., 2010), visual short-term memory (Boot et al., 2008), decision making (Green et al., 2010), multitasking (Green and Bavelier, 2006a), and multisensory integration (Di Luzio et al., 2021). Among action videogame (AVG) studies, first-person shooters (FPS) games – like Call of Duty, Counterstrike (CS:GO), Battlefield, and Fortnite – are centered on weapon combat using a first person perspective (i.e., the player looks at the scene through the eyes of the main character in the game), consequently requiring high flexibility, task switching skills, and rapid reaction times (RTs) (Colzato et al., 2010).

Apart from the direct impact on the specifically trained abilities, the success of a given intervention/training program occurs when people apply their previously accumulated experience-based skills to a new context or scenario (Thorndike and Woodworth, 1901; Tillberg, 2020). Such transfer of training/learning from one skill/ability to another is still a topic of discussion (Baldwin and Ford, 1988; Lieberman et al., 2014) as it is not yet clear whether exercising a specific skill can lead to an effect on abilities different from the practiced one (Gallagher et al., 2005; Morrison and Chein, 2011; Chooi and Thompson, 2012). The transfer of learning can be defined into a two-category model (Sala and Gobet, 2017) consisting in (i) *near transfer* learning: generalizing a skill to solve similar problems (e.g., once we have learned to tie a shoelaces, we will use this knowledge to tie all the shoelaces of the shoes we will buy during life, despite changing the thickness, length, or color of new shoelaces) (Alloway and Warner, 2006) and (ii) *far transfer* learning: use the knowledge to solve novel problems (e.g., a person who has learned the wind flow principles can transfer and use that knowledge to steer the sail on a sailboat). Moreover, recent studies have demonstrated beneficial transfer effects to brain functions that were not directly trained in the realm of video gaming itself, such as working memory or visuomotor control, which have been reported to occur even in older populations (Anguera et al., 2013; Latham et al., 2013) and possibly being transferrable to enhance performances in daily complex skills such as driving (Li et al., 2016).

Computerized virtual reality scenario are established in the context of training of various professional figures, for instance, airliner pilots using flight simulators recreating real scenarios (Haslbeck et al., 2014) or virtual trainings for surgeons (Boyle et al., 2011; Ahlborg et al., 2015). In this context, personalization of training regimen based on individual characteristics is crucial and it can be used to enhance specific cognitive functions both in healthy individuals and patients, with favorable outcomes in everyday life activities (Klingberg et al., 2005; Jaeggi et al., 2008; Alloway, 2012; Shinaver et al., 2014; Spencer-Smith and Klingberg, 2015). Specifically, tailored interventions have been frequently shown to outperform general approaches both in the clinical and no-clinical settings (Baker et al., 2015). In the case of videogames, personalization can also help in coping with aspects related to the game being either too easy or too difficult, resulting in a boring or frustrating experience, with potential adverse consequences at the research level, such as an increased risk of ceiling or floor effects (Lawrence, 2012). Furthermore, difficulty levels tailored at the individual pace – rather than on the general population sampling – could also ensure a greater control on the data collection and analysis. To the best of our knowledge, no previous studies have compared personalized or adaptive protocols to the standard in-game progression algorithms and their impact on game and cognitive abilities.

In the present study, we aimed at verifying whether a personalized/adaptive action videogame experience could elicit stronger benefits in terms of in-game performance and cognitive enhancement compared to the same videogame played according to standard, off-the-shelves parameters. We compared the in-game learning curves, as well as cognitive performance, of healthy participants following a training with a competitive FPS game called “Counter Strike: Global Offensive” (“CS:GO” hereafter; Valve and Hidden Path, 2012). A first group of participants played the “default” version of the game (Default CS:GO, “D-CS:GO”), whilst a second group was presented with a personalized adaptive training (Adaptive CS:GO, “A-CS:GO” from here on out). This allowed to carefully individualize the gaming sessions, providing equally entertaining and challenging trials both along the experiment period and across subjects. To monitor for the eventual transfer effects, participants were assessed for their cognitive profile before (T0) and after the experiment (T1: immediately after the end of the game period; T2: 3 months later). We hypothesized that (i) adaptive/personalized training might lead to a superior gaming performance than playing CS:GO using default parameters, and that (ii) the two training regimes would lead to a different modulation of cognitive abilities, with a higher performance for the adaptive group both in the short and long term.

## Materials and Methods

### Design and Participants

The study design included a baseline assessment (T0) consisting of a training session on the game modalities and features of CS:GO, a battery of cognitive tasks and an in-game assessment of the participants’ skills. After that, participants performed 15 daily gaming sessions, each lasting approximately 2 h for a minimum of 6 h per week, followed by a post-gaming in-game skills assessment and post-gaming cognitive assessment (T1). Follow-up cognitive assessment was repeated 3 months after the end of gaming sessions (T2).

For this study, the inclusion criterion was to be classified as AVGP, therefore every candidate compiled a questionnaire to quantify the time spent playing videogames (Green et al., 2017). A total of 21 young healthy were identified as AVGPs (action videogame experience ≥ 3–5 h per week during the last year) and were enrolled. None of the participants reported prior experience with a competitive FPS game. Each subject was randomly assigned to either one of two groups: the “A-CS:GO” group, playing a customized version of the videogame (11 subjects, four females and seven males; 24.2 ± 3.1 years), and the “D-CS:GO” group (10 subjects, two females and eight males; 24.1 ± 2.3 years), playing the off-the-shelves version of the game. Details on the progression algorithm are reported in a dedicated paragraph below.

Gaming was performed on a 19-inches screen located 80 cm away from the subject, with the prohibition of playing at home with any videogames. All participants had no history of neurological or psychiatric disorders, assessed via the Mini-International Neuropsychiatric Interview (Sheehan et al., 1998). Each participant provided a written informed consent. Study was approved by the local ethical committee.

### First-Person Shooter Training Structure

CS:GO is an FPS game which differs from classic action games due to its competitiveness, as it requires the acquisition and the continued improvement of tactic and strategic skills related to specific game battlefields played over timed rounds. Indeed, while in a classic FPS game, the gamer has multiple scenarios to play and often switches scenario every few minutes of gameplay, CS:GO is played on a limited number of maps, thus requiring a deep knowledge of team-play strategies and fine 3D navigation. Thus, the game constantly demands high visuomotor coordination, fast RT, optimal perceptual skills, good planning behavior, high flexibility, and appropriate inhibition. For these reasons, and because it can be played cooperatively in competitive matches, the game is currently used in the professional electronic sports contexts^1^.

For the present study, the game was played in the offline modality (Local Area Network), allowing to play against artificial intelligence (AI) guiding computer-controlled opponents (“Bots” hereafter). The difficulty level of each bot is defined by a combination of skills, including speed, aiming accuracy with different weapons, cooperative behavior, aggressiveness, weapons at their disposal, and “swarm” intelligence (i.e., ability to coordinate with other bots in complex scenarios requiring cooperation). These characteristics are defined by the game engine and grouped to define four difficulty levels (Easy, Normal, Hard, Expert).

Introduction to the game was given throughout the first day with a 2-h session, allowing players to become familiar with the game commands and interface. Team-Deathmatch game modality was chosen for 15 daily gaming sessions, consisting of four rounds × 20 min each. In this modality, players must gain the highest possible scores, increasing their rate of survival while maximizing the number of bots being defeated at each round. Game mechanics included the player joining one of two teams and one of the four predefined maps, with the aim of increasing the CS:GO score of his/her team. Neither the same Team nor the same map could be chosen more than twice in a row to increase complexity and variability.

Each session lasted approximately 2 h, with participants playing a 10′ warm-up round without any performance monitoring, then training for 40′ (2 rounds × 20 min each), resting for 10′, and lastly, playing for an additional 40′ (2 rounds × 20 min each). In each session, participants joined one of the two teams (player + 4 AI-guided allies) with the aim of overpowering the opposite team (five bots).

Participants joined each gaming session on separate desktop PCs without the possibility of communicating with each other. After each round, the ratio between kills and death (K/D score) was registered by investigators. Gaming sessions were carried out using a dedicated desktop PC, equipped with: high performance dedicated graphic card = ATI Radeon 4GB, RAM = 8GB, 21^″^ LCD monitor [Frame Per Second (FPS) = 60 Hz], headphones, mouse, and a keyboard.

### Adaptive Algorithm

For the A-CS:GO group, the difficulty level (ranging on a scale from 1 to 24) was manually set up by the experimenter. An *ad hoc* algorithm was conceived for the study, allowing for the manipulation of the difficulty of each gaming session according to the participants’ performance on the previous gaming session. The personalization was done by creating a set of custom bots characterized by almost 100 combinations of in-game skills, specifically related to:

(i) **Aim focus:** bot’s RT and accuracy when focusing on a new target (i.e., similar to accuracy of saccades and gaze in humans);
(ii) **Aim focus interval:** time required to re-focus on a new target (i.e., in human executive functions terms, this could be ascribed to Flexibility/Switching ability);
(iii) **Aim focus decay:** duration of aim on a given target before engaging in alternative routines (i.e., sustained attention capacity);
(iv) **Reaction time:** RT to an event, specifically to the appearance of an enemy on the screen, new weapons available, change of strategic priority (i.e., similar to a general RT in humans);
(v) **Team working:** ability to cooperate with other teammates, e.g., protect wounded bots, target enemies closer to teammates;
(vi) **Aggressivity:** tendency to attack over retreat, chase enemies down instead of holding a safe position, and wait for back-up;
(vii) **Proficiency with a specific weapon:** ability with specific weapons (knife, gun, uzi, rifle, and shotgun); and
(viii) **Speed:** movement speed (walk, run, and spin).

Resulting bots were then combined in teams of five or more components by balancing each bot’s skill to obtain a certain level of difficulty (e.g., *Elite team* = 5 bots with good proficiency in each weapon, high aiming skills, fast RT, high cooperative behavior, high proactiveness, high speed; *Medium team* = 2 bots at Elite level, two bots with average aiming and RTs, one bot with poor aiming/RT and good proficiency in only 1–2 specific weapons, medium proactiveness, medium speed; *Low-tier team* = 4 bots with average aiming and RT, proficiency in only 1–2 specific weapons, one bot with low aiming and RT, proficiency in only one weapon, low cooperation, low proactiveness). The combination of bots resulted in an *ad hoc* difficulty level scale from 1–24, instead of the four-levels scale used in the standard game (Easy, Medium, Hard, and Expert). The 24 levels were obtained by (i) combining bots of different skills in (ii) teams of five or more bots (six from level 10 onward), by (iii) equipping them with different weapons, and by (iv) selecting maps of different sizes.

**FIGURE 1:**
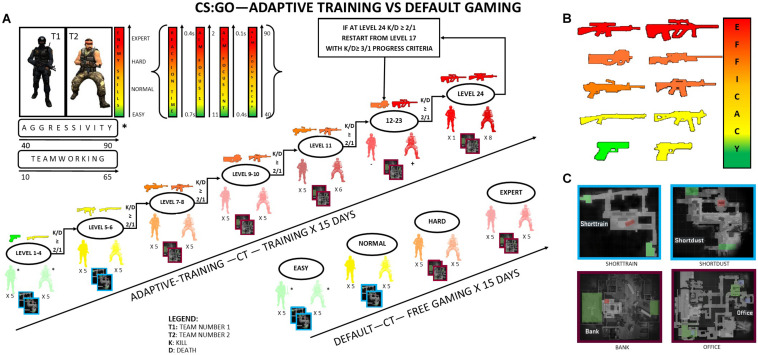
Scheme of the experiment protocol. **(A)** At the beginning of the game, the player could join one of the two teams available in the game: Team 1 or Team 2. The A-CS:GO group played a customized game in which the progression in the game was established on the bases of the Death/Kill (K/D) ratio. If a player reached the K/D ≥ 2/1 for two consecutive rounds, he/she could proceed into the next level. Once reaching level 24, participants restarted from level 17 and could proceed to the next level only if he/she was capable of reaching a K/D ≥ 3/1. The D-CS:GO players could choose their difficulty level between four standardized games at the beginning of each round (*players joined a team and faced some enemies. The number of team bots and enemy bots was customized, as well as their skills^∗^). **(B)** Weapons’ efficacy was set from the less (green–yellow) to the most (yellow–orange) dangerous on the bases of the extent of the capacity to cause damage, firing rate, and reload velocity. **(C)** Four different maps were available in the game. The smallest and easiest maps (Shorttrain and Shortdust) characterized the first levels of the A-CS:GO group and the Easy/Normal modality in the D-CS:GO group, whereas larger maps (Bank and Office) were proposed to challenge subjects in the A-CS:GO group and in Hard/Expert modality of the D-CS:GO group.

The A-CS:GO game progression was governed by a 2:1 rule: the level was manually increased by an investigator when the numbers of kills doubled individual’s death (i.e., K/D ≥ 2) for two consecutive rounds. The requirement of completing two rounds at K/D > 2 was aimed at ensuring that the increase in K/D ratio was reflective of a genuine increase in the participant’s skill. Participants in the A-CS:GO group progressed through the 1–24 difficulty levels, constantly trying to maintain at least a K/D > 2. If a player reached the 2:1 criterion at the last available level (#24), he/she restarded from level 17 and challenged with a new K/D criterion of 3:1 (Figure 1). Notably, the initial difficulty level was set by an investigator based on the performance at the first two rounds: players started their first round at level 4, if the 2:1 criterion was not met, the difficulty level was lowered up to level 1. If the player reached the K/D ≥ 2/1 at level 4, he/she was then tested at level 6; if the 2:1 criterion was satisfied again, the training session started at level 7; if not, training started at level 5.

Importantly, the different difficulty system between D-CS:GO and A-CS:GO did not allow for a direct comparison of the K/D levels across groups. Given that each participant in the A-CS:GO group was moved to the next level in case he/she achieved a 2:1 K/D, resulting raw K/D ratios were not representative of individual performance. Therefore, a composite score obtained by multiplying K/D values for each session by their corresponding difficulty level was computed (e.g., K/D = 2.3, difficulty = 16; corrected K/D = 2.3 × 16 = 36.8). In order to calculate the same score for the D-CS:GO group, the four difficulty levels (Easy, Medium, Hard, and Expert) were mapped to corresponding ones in the A-CS:GO difficulty system: (Easy = level 1; Medium = level 5; Hard = level 7; and Expert = level 10).

Participants in the D-CS:GO group were allowed to freely decide their game difficulty level, choosing between Easy, Medium, Hard, or Expert (Figure 1). However, participants were still asked to meet the requirement of a K/D > 2, i.e., they were aware of the nature of the training and the goal of improving their in-game performance as much as possible within the 3 weeks of training period.

### In-Game Performance Assessment

**FIGURE 2:**
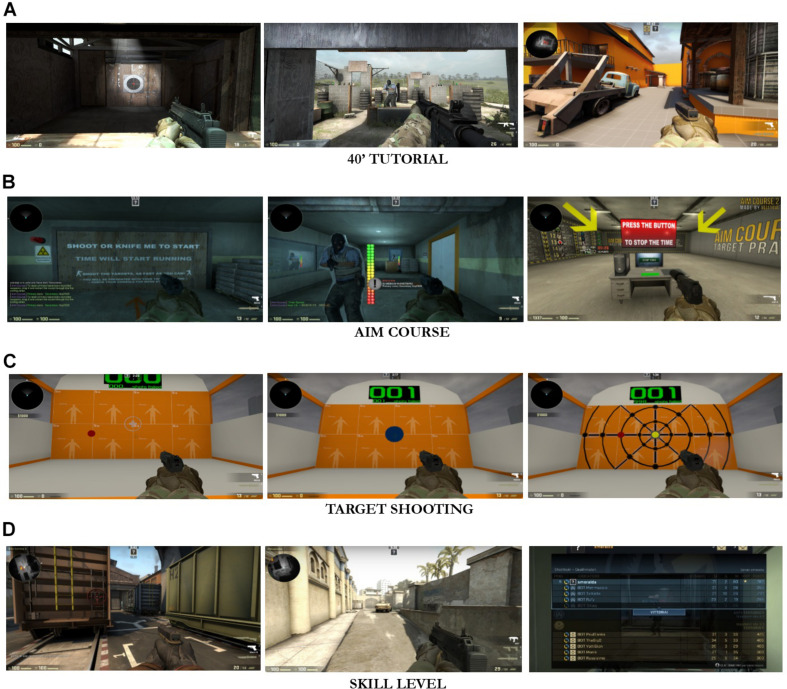
Game skills assessment. **(A)** During the initial assessment, each player completed 40′ of tutorial to understand the game controls and the environment. **(B)** At T0 and T1, subjects completed the Aim Course time-constrained task. The times required to complete the Aim Course 1 and Aim Course 2 Maps were registered. **(C)** At T0 and T1, the participants engaged in various shooting tasks and accuracy was recorded. **(D)** At the initial assessment, the A-CS:GO group was further assessed on a fourth task, aiming to define the level of player game-ability. This task established the player’s starting level.

Both the D-CS:GO and A-CS:GO groups performed an initial evaluation of their FPS skills and were tested on two sets of tasks: (i) a training course involving navigating a map while shooting at targets and (ii) a set of firing range simulations assessing various types of coordinated visuomotor skills relevant for aiming (Figure 2).

#### Tutorial

An initial 40-min game tutorial was carried out to ensure a complete understanding of the game dynamics, commands, and interface (Figure 2A).

#### Training Course

Two time-constrained training courses (Figure 2B; Aim Courses 1 and 2) were tested: players had to complete a guided path as fast as possible while shooting randomly at the appearance of the enemies’ silhouettes. Completion time was measured, representing the ability to navigate a 3D environment under pressure as well as pure aiming skills.

#### Firing Range

Participants were assessed on their firing accuracy in a virtual shooting gallery, completing six aiming tasks with a handgun (Figure 2C):

(i) *“Static” task* = participants shoot 50 targets appearing at the center of the visual field for 1 s;
(ii) *“Linear” task* = participants shoot 50 targets appearing on the screen for 2 s and moving in linear trajectories horizontally or vertically at two different speed levels (linears 1 and 2);
(iii) *“Static* + *linear” task* = a combination of the two previous tasks;
(iv) “*Angles*” task = participants shoot 50 targets moving diagonally from the center of the screen; and
(v) *“Reflex” task* = participants shoot to target moving randomly from the center of the screen;

Participants in both the D-CS:GO and A-CS:GO groups were tested with the same game assessment at T0 and T1.

#### Gaming Skills/Ability

Players played a game for 20 min on a game level (Figure 2D) to establish their gaming skills, calculated by the ratio between the number of enemies killed and number of defeats.

### Cognitive Assessment

The entire cognitive battery of tasks was assessed at T0 and T1, while at T2, only a reduced battery of tasks showing a significant effect at T1 compared to T0 was administered. Tasks were performed via the E-prime 2.0 software (Psychology Software Tools Inc.^2^; Schneider et al., 2012) on a Windows laptop PC. The battery-tasks measured multiple cognitive domains such as visuo-spatial abilities, attention, inhibition, and switching (for details, see Supplementary Material). Moreover, *ad hoc* visuo-spatial tasks were administered to assess the impact of videogames as previously shown by numerous studies (Green and Bavelier, 2003, 2006a,2006b, 2007) and verify near, moderate, and far transfer. In fact, FPS games can have a direct impact on basic skills, such as attention and filtering [due to the continuous requirement to discriminate distractors among salience target (Green and Bavelier, 2006a)], hand-eye coordination and contrast sensitivity [(greater in AVGPs respect to NVGPs) (Green and Bavelier, 2012)], and even on executive functions (i.e., cognitive flexibility, planning, and decision making; Basak et al., 2011). Therefore, tasks were categorized as “Far,” “Moderate,” and “Near” transfer based on the nature of the videogame implicated in our study and considering precedent findings (Green and Bavelier, 2003, 2006a,2006b).

#### Near Transfer

An FPS game mainly recruits attentional and visuospatial skills. We measured these cognitive functions by means of a series of tasks: Serial RT (Robertson, 2007), Mental Rotation (Cooper and Shepard, 1973), Useful Field of View (UFOV) (Feng et al., 2007), Visual Search (Treisman and Gelade, 1980), Flanker (Green and Bavelier, 2003), and Attentional Blink (Raymond et al., 1992).

#### Moderate Transfer

To measure inhibition abilities, we used these tasks: Preparing to Overcome Prepotency (POP) (Rosano et al., 2005), Letter No-Go (Thorell et al., 2009), and Global-Local Features (Navon, 1977) tasks.

#### Far Transfer

We verified changes in higher cognitive functions such as flexibility, reasoning, and memory using Raven’s Advanced Progressive Matrices (Raven et al., 1998), Sandia Matrix Task (Matzen et al., 2010), Digit Span (Wechsler, 1981), and Change Localization Task (Luck and Vogel, 1997).

### Statistical Analysis

Analyses were carried out using the SPSS version 16, SPSS Inc., Chicago, IL, United States. Three main analyses were carried out: (i) in-game progression/performance based on K/D at T0–T1; (ii) changes at in-game assessment based on training courses and firing range simulations measured at T0–T1; and (iii) transfer of cognitive abilities measured with cognitive tasks at T0–T1–T2. Subjects were not allowed to play videogames at home between T0 and T1, while no specific task was assigned to the participants between T1 and T2.

#### In-Game Progression (K/D Ratio) Analysis

First, the raw K/D ratio (uncorrected K/D ratio) of each round was multiplied by the level of difficulty of the same session (corrected K/D ratio). A quadratic regression was conducted considering the game performance during training (60 rounds) to test if Time significantly predicted the participants’ corrected K/D ratio.

Second, we calculated the average uncorrected and corrected K/D ratio over the four rounds played each training day. A preliminary analysis was conducted on the first training day, to verify the differences between the A-CS:GO and D-CS:GO groups at the beginning of the training. Therefore, a Repeated Measures Analysis of Variance (ANOVA_RM_) was conducted on the corrected and the uncorrected K/D ratio, to verify the performance differences between groups during the training period with Time (15 levels: from Day 1 to Day 15) as within-subjects factor and Group (two levels: A-CS:GO and D-CS:GO) as between-subjects factor. Again, a quadratic regression was conducted to test if Time significantly predicted the participants’ game performance during the training days (15 days). A *Post hoc* power analysis was performed using the G-Power software (Faul et al., 2007) in order to calculate the statistical power of the ANOVA results.

#### In-Game Assessments Analysis

An ANOVA_*RM*_ was performed to verify game ability changes using Time (2 levels: T0 and T1) and Group (2 levels: A-CS:GO and D-CS:GO) as factors.

#### Transfer on Cognitive Abilities Analysis

Cognitive tasks were administered to both A-CS:GO and D-CS:GO groups before (T0), immediately after (T1), and 3 months after the end of the experimental procedure (T2). Due to scheduling conflicts, only 19 subjects out of 21 gamers underwent T2 re-testing. In order to verify significant long lasting changes in gamers between the pre, post, and follow up assessments, aANOVA_*RM*_ was conducted with Time (three levels: T0, T1, and T2) and Group (two levels: A-CS:GO and D-CS:GO) as factors.

## Results

### In-Game Progression Results

**FIGURE 3:**
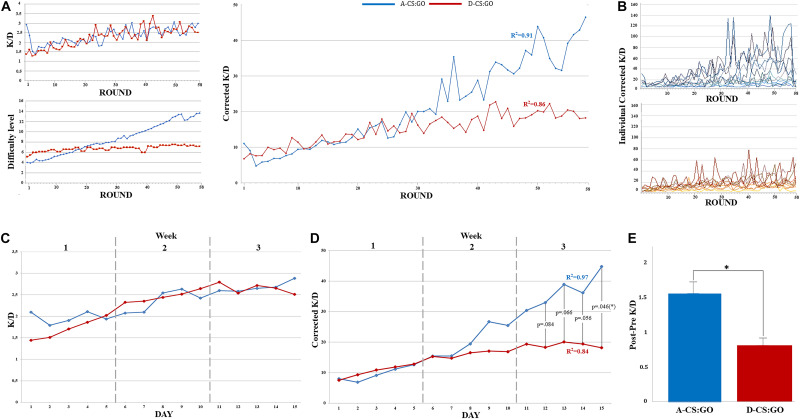
Graphical depiction of the in-game progression for both A-CS:GO and D-CS:GO groups. **(A)** uncorrected K/D ratio, difficulty levels and corrected K/D ratio of the training rounds. **(B)** Individual corrected K/D ratio changes during training rounds. **(C)** K/D ratio during the training days. **(D)** Corrected K/D ratio during the training days. After an initial equivalent performance between A-CS:GO and D-CS:GO groups, the corrected K/D ratio progressively increased in the A-CS:GO group and the performance of the A-CS:GO group becomes significantly higher than the CS:GO group performance. (*p < 0.05 – higher performance in the A-CS:GO group). E: Pre-training corrected K/D was subtracted from post-training performance for both groups, highlighting a significant improvement of performance after the A-CS:GO training (*p < 0.05).

Figure 3A illustrates the uncorrected K/D ratio, the difficulty levels and the corrected K/D ratio of the training rounds. Regression analyses indicated that Time (i.e., number of training sessions) predicted the corrected K/D ratio in the A-CS:GO (*R*^2^ = 0.91; β = 0.51, *p* < 0.001) and in the D-CS:GO group (*R*^2^ = 0.86; β = 1.70, *p* < 0.001). Figure 3B shows the corrected K/D ratio of each participant.

Similar to that shown in each round, the profile of the uncorrected K/D performance did not differ between trained groups during the training days (Figure 3C). The Mauchly’s test of the ANOVA_RM_ showed a violation of the assumption of sphericity ($\chi_{\left(104\right)}^{2}$ = 189.93, *p* < 0.001) and a Greenhouse-Geisser estimation of sphericity was used to correct the degrees of freedom (ε = 0.37). The analysis revealed a significant effect of Time (*F*_(5.27, 100.18)_ = 7.76, *p* < 0.001) and no interaction between Time and Group. On the other hand, by correcting the K/D ratio with the difficulty level and conducting ANOVA_RM_, significant difference was highlighted between A-CS:GO and D-CS:GO groups during the training days (Figure 3D). Again, Mauchly’s test indicated that the assumption of sphericity was violated, ($\chi_{\left(104\right)}^{2}$ = 471.09, *p* < 0.001) and the degrees of freedom were corrected by using Greenhouse-Geisser estimates of sphericity (ε = 0.16). Results showed a significant effect of Time (*F*_(2.32, 44.08)_ = 13.25, *p* < 0.001) and a significant interaction of Time x Group (*F*_(2.32, 44.08)_ = 4.22, *p* = 0.017) with an higher performance in the A-CS:GO. Input parameters of the power analysis were: effect size f: 0.4 (calculated from the significant interaction of Time x Group ANOVA_RM_ analysis: *d* = 0.4 90% CI [0.14–0.67]); groups: 2 (A-CS:GO and D-CS:GO); measurements: 15 (training days) alpha: 0.05; non-sphericity correction ε: 0.16. We calculated a statistical power of 0.93 (1-β error probability) to discriminate significant differences during the training period and between different conditions (Noncentrality parameter λ: 90.97; Critical *F*: 3.09; degrees of freedom: 2.24, 42.56). Figure 3E shows Post-Pre performance difference. The results of the *t*-test highlighted an higher corrected K/D ratio in participants exposed to A-CS:GO: (*t*_(19)_ = 4.47, *p* < 0.05).

### In-Game Assessment Results

A general effect of Time (T0 and T1) was observed for all game tasks (Table 1). The *Aim Course 1* task analysis revealed a main effect of Time (*F*_(__1_,_19__)_ = 17.758, *p* < 0.001), such as that both groups (A-CS:GO and D-CS:GO) showed a faster completion time at the post-training assessment compared to the pre-training assessment (*t*_(__18__)_ = −4.27, *p* < 0.001). The same pattern was observed for the *Aim Course 2* task (*F*_(__1_,_19__)_ = 15.642, *p* = 0.001), with higher performances achieved after training in both groups (*t*_(__18__)_ = −4.05, *p* < 0.001). No interaction between Time and Group was found, although a trend for a better performance in the A-CS:GO group compared to the D-CS:GO group was observed, with the A-CS:GO group fastening their time of task execution (−46.09 s between T0 and T1) more than the D-CS:GO group (−31.5 s between T0 and T1).

**TABLE 1:** Group Means and Standard Deviation (SD) for the pre (T0) and post (T1) game assessment.

**Task**	**MEASURE**	**Means (*SD*) adaptive T0**	**Means (*SD*) adaptive T1**	**Means (*SD*) default T0**	**Means (*SD*) default T1**
Aim Course 1	Time	02:53:27 (*00:55:27*)	02:06:16^a^ (*00:24:56*)	02:23:12 (*00:45:53*)	01:53:24^a^ (*00:22:04*)
Aim Course 2	Time	03:21:33 (*01:09:46*)	02:36:44^a^ (*00:33:37*)	02:47:48 (*00:55:19*)	02:14:36^a^ (*00:29:39*)
Static Target	Hits	34.55 (*8.32*)	41.18^a^ (*5.98*)	36.95 (*10.26*)	45.00^a^ (*3.84*)
	Fails	15.82 (*7.29*)	9.32^a^ (*5.86*)	15.80 (*13.37*)	5.20^a^ (*3.91*)
Linear Target 1	Hits	31.68 (*12.14*)	38.50^a^ (*9.26*)	34.60 (*9.99*)	41.55^a^ (*4.54*)
	Fails	37.59 (*20.79*)	21.23^a^ (*11.26*)	48.75 (*33.65*)	24.20^a^ (*9.33*)
Linear Target 2	Hits	28.21 (*10.23*)	28.59^a^ (*10.05*)	31.04 (*9.72*)	34.62^a^ (*14.88*)
	Fails	28.42 (*12.48*)	21.34 (*8.96*)	36.38 (*31.15*)	45.83 (*10.12*)
Static + Linear Target	Hits	35.73 (*6.75*)	41.05^a^ (*6.01*)	37.05 (*7.43*)	41.60^a^ (*4.99*)
	Fails	21.09 (*7.81*)	13.36^a^ (*5.97*)	27.60 (*18.37*)	17.65^a^ (*8.19*)
Angles Target	Hits	71.27 (*7.73*)	76.36^a^ (*14.29*)	72.10 (*10.76*)	79.65^a^ (*8.79*)
	Fails	938.00 (*8.72*)	915.91^a^ (*63.28*)	934.85 (*11.82*)	925.70^a^ (*9.98*)
Reflex Target	Hits	27.05 (*5.65*)	30.95 (*6.74*)	26.75 (*6.95*)	28.55 (*5.93*)
	Fails	22.50 (*5.80*)	19.05 (*6.74*)	23.25 (*6.95*)	21.45 (*5.93*)

*^*a*^Significant higher performance at T1 than T0, italic values means (*p* < 0.05), SD is for “Standard Deviaton”.*

In the *Static, Linear 1, Linear 2, Static* + *Linear*, and *Angles* aim tasks, a main effect of Time was also found (respectively: *F*_(__1_,_19__)_ = 28.384, *p* < 0.001; *F*_(__1_,_19__)_ = 18.621, *p* < 0.001; *F*_(__1_,_19__)_ = 6.618, *p* = 0.019; *F*_(__1_,_19__)_ = 26.239, *p* < 0.001; *F*_(__1_,_19__)_ = 22.927, *p* < 0.001). Trained subjects missed less targets in the post-game assessment (respectively: *t*_(__18__)_ = −5.11, *p* < 0.001; *t*_(__18__)_ = −4.33, *p* < 0.001; *t*_(__18__)_ = −4.15, *p* < 0.001; *t*_(__18__)_ = −4.21, *p* < 0.001), simultaneously entailing higher number of hits in all tasks but *Angles* (respectively: *t*_(__18__)_ = 5.90, *p* < 0.001; *t*_(__18__)_ = 6.42, *p* < 0.001; *t*_(__18__)_ = 3.51, *p* = 0.002). No significant interactions between the Time and Group were found. Finally, no significant effects were observed for the *Reflex* task.

### Long-Term Cognitive Transfer: Effect of Training

**FIGURE 4:**
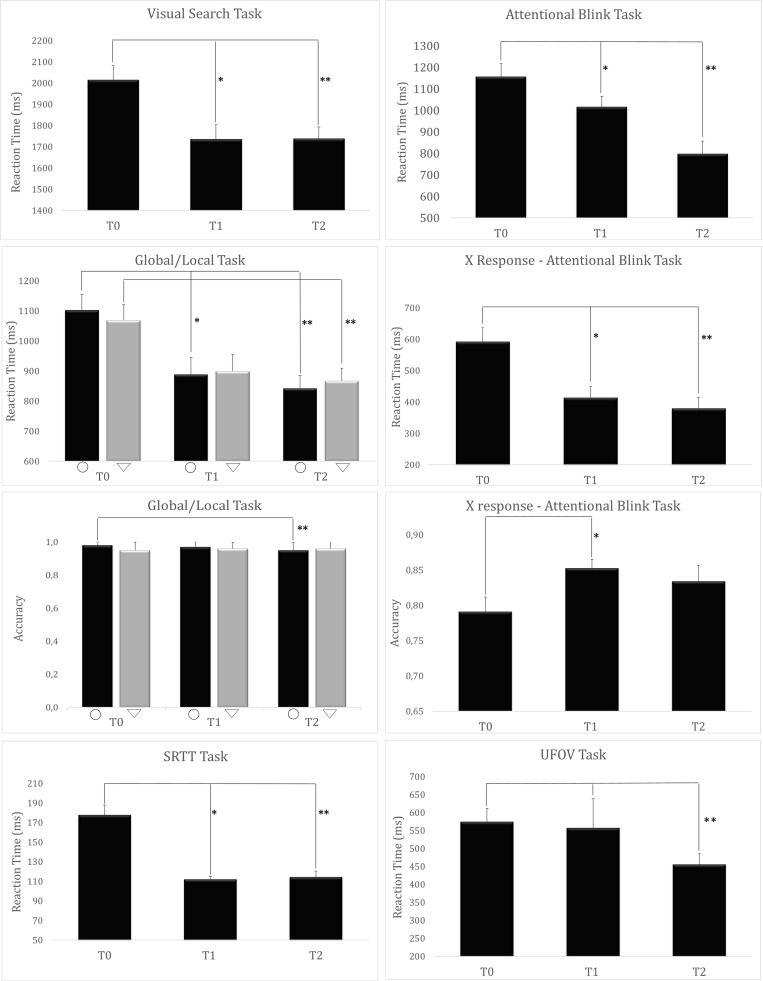
Long Term Cognitive Transfer results. Graphical depictions of the significant effects observed between cognitive assessments (^∗^significant difference between T1 and T0; ^∗∗^significant difference between T2 and T0; Global/Local Task: ∇: Global Features ○: Local Features).

No differences between the two groups have been observed in the cognitive tests performance analysis at T0 (*p* > 0.05). ANOVA_*RM*_ revealed a main effect of Time for the subjects’ RTs in *Visual Search* (*F*_(__2_,_16__)_ = 7.848, *p* = 0.004), such as that participants responded faster at T1 than at T0 (*p* = 0.009). Such effect remained stable even at T2 compared to T0 (*p* = 0.002). No difference between T1 and T2 was observed. Concerning the *Global features* Accuracy *of Global Local Task*, a main effect of Time was found (*F*_(__2_,_16__)_ = 4.483, *p* = 0.028) with a lower performance at T2 than T0 (*p* = 0.024). At the *Global-Local* Task, a main effect of Time for RT to the *Global features* was observed (*F*_(__2_,_16__)_ = 12.004, *p* < 0.001). Responses were faster at T1 compared to T0 assessment (*p* = 0.002). After 3 months, reaction remained faster than the T0 (*p* < 0.001). For *Local features* RT, a main effect of Time was found (*F*_(__2_,_16__)_ = 8.929, *p* = 0.002). Subjects were faster at T2 compared to T0 (*p* = 0.002), but not at T1. In the *Attentional Blink* task, a main effect of Time was also observed (*F*_(__2_,_16__)_ = 34.143, *p* < 0.001). Subjects were faster in responding to the target stimulus at T1 compared to T0 (*p* = 0.003). Higher speed was further observed at T2 compared to T0 (*p* < 0.001). Similarly, an effect of Time of RT to the letter X was observed (*F*_(__2_,_16__)_ = 29.413, *p* < 0.001), with a faster response at T1 and T2 compared to T0 (*p* < 0.001 for both comparisons). A main effect of Time (*F*_(__2_,_16__)_ = 5.555, *p* = 0.015) was observed considering the participants’ accuracy in detecting the target letter X, such as that the participants were more accurate at T1 than T0 (*p* = 0.01). For what concerns the *SRTT* task, RTs lowered as a function of Time (*F*_(__2_,_16__)_ = 20.069, *p* < 0.001), with better performances at T1 and T2 compared to T0 (*p* < 0.001 for both comparisons). As per the *UFOV* task, Time exerted a significant effect in terms of overall faster RTs (*F*_(__2_,_16__)_ = 7.511, *p* = 0.005), leading to higher performances at T2 compared to T0 (*p* = 0.004). All effects are reported in Figure 4.

## Discussion

A personalized, adaptive version of a competitive FPS videogame was compared with its standard version, revealing a greater improvement in videogame performance for participants exposed to the former. Additionally, cognitive benefits were detected after 15 sessions of intensive video gaming (∼30 h) which were maintained up to 3 months after the end of the study in both experimental groups. Relevance for cognitive enhancement applications, potential implications for the professional videogame industry as well as on the impact of videogames on brain and cognition are discussed.

### Individualized Video Gaming

Previous literature reported a beneficial effect of individualized gaming task on clinical populations (Davis et al., 2018). Thus, we hypothesized and expected the achievement of better videogame and cognitive performances in the adaptive trained group of our healthy subjects’ sample. Videogames provide entertainment via immediately rewarding experience, substantiated by neuroimaging findings suggesting a pivotal role of the striatum – part of the neural reward system – during video gaming (Lorenz et al., 2015; Momi et al., 2018b). This mechanism might be capitalized to the exploitation of personalized video gaming to obtain potential benefits for cognitively impaired populations as well as in the prevention of cognitive weaknesses (Mishra et al., 2016). Furthermore, to decrease the probability of drop-outs and further guarantee adequate long-lasting motivation and game competence, it is critical to create individualized game environments (Ryan et al., 2006). We did not observe significant drop-outs, confirming the validity of the adaptive algorithm, which ensured the proper balance between the increment of difficulties and positive reinforcement and entertainment (Grodal, 2000).

In particular, “close-loop tasks” might be essential in order to accelerate and reinforce the progression on videogame, as well as in everyday life skills (Sitaram et al., 2017). By receiving feedbacks on their performance, participants can better understand where it is better to allocate their effort, thus maximizing their performance on a specific goal (Mishra et al., 2016). During gaming, rewards are provided through visual or auditory feedbacks, aiming to maximize motivation (Bavelier et al., 2012; Ahlborg et al., 2015). Accordingly, the constant monitoring over measures of performance and the corresponding ongoing adjustments of gaming parameters enabled the distinction between two differential learning curves among our experimental groups: a proficiency-based profile for the A-CS:GO group and a fixed time bound practice for the D-CS:GO group. Proficiency based learning is a feasible component that concerns, for example, the way humans learn at school or new skills in general, giving the possibility to progressively overcome consecutive gaps for optimal learning (Snyder et al., 2009). For example, it has been shown that future surgeons learn better surgical skills if, during a virtual reality training program, the difficulty of simulated operations becomes progressively harder (Gallagher et al., 2005).

An interesting finding concerns the different trajectory of the learning curve across the training sessions, with the A-CS:GO group progressively outperforming the D-CS:GO group (Figure 3). In the adaptive modality, the number of enemies increases and allies decreases after the 10th level. In the meantime, available maps rise for their complexity. Starting from level 15, the number of allies keeps on decreasing (four allies vs. eight enemies bots) and ensures that the player’s ability continues to increase until the end, even after the participants have already explored all the available maps and weapons. This suggests that players reach a discrete knowledge and confidence with all available scenarios and videogame options, becoming capable of capitalizing such knowledge to face and overcome new challenging situations. As shown by trendlines and regression analysis (Figure 3D), training time is a strong predictor for the game performance in both groups. On the other hand, a stronger prediction is noticeable in the A-CS:GO group, suggesting the usefulness of a proper individualized – and relatively short in time – training in order to reach a better performance in complex tasks. Furthermore, it demonstrates how the increasing difficulty of an adaptive training, based on the player’s specific characteristics, could be a better setting than a free game.

Regarding the game assessment results, both groups improved their performance in the target shooting tasks. All participants became accurate in hitting more targets and making less mistakes. Moreover, a trend toward the significance of a decreased execution time in the Aim Course task was found for the A-CS:GO. In order to defeat the growing number of enemies, A-CS:GO participants had to quickly adapt themselves and adopt different gaming styles, compared to those of the D-CS:GO participants. Fast moving is a requisite for the A-CS:GO players: they have to hit the enemies and quickly move to another playing area in order not to be hit back. This is exactly what happened in the Aim Course task: the faster the player hits the shapes of the enemies, the faster he can move to another area and finish the course.

Recently, video gaming is no longer considered just an entertaining activity for some people, but it has become an opportunity for a real career. More and more people are investing their future in this activity, participating in competitive tournaments that reward large sums of money, and its activity is so widespread that professional videogamers are also called electronic athletes (Parke et al., 2005). The economic impact is enormous, so much that many brands have invested to advertise during professional tournaments and support players (Bányai et al., 2019). The reduction of the learning curve has an interesting implication for those who wish to approach with professional video gaming. Decreasing the acquisition time of a skill game could in fact lead the player to achieve goals faster and it is possible to hypothesize that an individualized protocol – such as the one developed in our studio for CS:GO – could be used in other FPS games, which are the ones most often used in professional tournaments.

### Cognitive Transfer

The hypothesized effect of a better performance of the A-CS:GO within the gaming environment has been found, confirming previous findings on how intensive exercise produces a marked improvement in the training task (Gallagher et al., 2005; Morrison and Chein, 2011; Chooi and Thompson, 2012; Mishra et al., 2016), but similar cognitive changes between the A-CS:GO and D-CS:GO groups observed at T1 and T2 assessments open additional questions related to video gaming and effective cognitive transfer.

According to Green and Bavelier, action videogame playing can enhance the ability of an individual to learn new tasks (Bavelier et al., 2012), a phenomenon defined as “*learning to learn*” (Harlow, 1949; Kemp et al., 2010). Specifically, AVGPs are considered more capable to extract regular patterns from different scenarios and suppress irrelevant information from distractors (Föcker et al., 2018). These benefits are expected to be generalized to other tasks sharing some of the structure or have similarities with the videogame (*near and moderate transfer*), whereas, they generally fail to expand to more distant cognitive processes (*far transfer*). In our study, we found cognitive benefits in both the A-CS:GO and D-CS:GO groups after the game training and these benefits were maintained in the follow-up assessment performed 3 months after the end of the experiment. This result suggests the possibility of obtain a long-lasting improvement following an FPS videogame training, with potential implications even for clinical applications. Regarding *near and moderate transfers*, we found an enhancement of cognitive skills directly stimulated by the videogame, specifically measured via the *Visual Search, UFOV, Attentional Blink, Global/Local, and SRTT* tasks, and especially for the RTs which were improved after game training. These tests are used to investigate the ability of the visual system to allocate attentional resources, inhibition skills, and visuo-motor learning. The effects observed in the present study align with previous literature reporting a transfer effect on the visual-attentive and visuospatial functions implicated during video gaming, especially in shortening the RT observed at T1 (Greenfield et al., 1994; Green and Bavelier, 2003; Castel et al., 2005), also showing the persistance of the effect 3 months after the end of the training (T2).

For instance, subjects with attention deficit hyperactivity disorder (ADHD) show slower RTs in complex attentional tasks (Gualtieri and Johnson, 2006). In the same patients, videogame-interface trainings have been used to modify attentional abilities and executive functions that are typically altered in this neuropsychological disorder (Davis et al., 2018). Moreover, it has been demonstrated that subjects with dyslexia have a larger attentional blink and reading speed inefficiency (Visser et al., 2004). Action videogame training has been proved to be capable to improve children’s reading speed (Franceschini et al., 2013). Recently, deficits in switching abilities have also been reported in Parkinson’s disease (Hélie and Fansher, 2018) for which the use of videogames have been recently used, improving their performance in activities of daily living and motor coordination (Pompeu et al., 2012). Moreover, in patients with multiple sclerosis, protocols combining video gaming with physical exercise and movement (“exergames”) have been applied, giving positive effects in higher cognitive functions, such as attention and executive functions (Prosperini et al., 2015; Lavorgna et al., 2018).

Interestingly, a recent investigation by our group has shown how playing CS:GO in its “default” modality might induce acute and long-lasting changes in brain structures (e.g., cortical thickness, volume) relevant for the currently observed cognitive effects. For instance, in one study, long-term morphovolumetric changes in the pulvinar were found (Momi et al., 2018b). The pulvinar is strongly connected with the occipital cortex and it has been shown to be involved in cognitive functions such as selective attention (Shipp, 2004), multisensory integration (Stein and Stanford, 2008) inhibition (Mitchell, 2015), and spatial seeking (Fischer and Whitney, 2009). Remarkably, all the aforementioned cognitive functions are consistently stimulated by an FPS where players are asked to identify salient targets (e.g., enemies) among distractors (e.g., teammates, neutral players), while constantly filtering irrelevant information. After an FPS training, a long-term increase of brain cortical thickness in the somatosensory, parahippocampal, and superior parietal lobule areas has been found (Momi et al., 2018a). These anatomical structures have been linked to navigation processing, spatial knowledge in 3D environments, visual-spatial skills, visuomotor attention, motor execution, inhibition, and saccadic eye movement (Stephan et al., 1995; Mort et al., 2003; Caplan et al., 2006; Ekstrom and Bookheimer, 2007; Naito et al., 2008; Rauchs et al., 2008; Asscheman et al., 2015) and might likely represent the neural bases of our near and moderate transfer effects. It is reasonable to assume that the application of an adaptive, personalized version of CS:GO, that is more effective for cognitive improvement, could lead to even stronger brain changes in pertinent regions. More in general, the overlapping enhancement of cognitive abilities across the two groups seems to fit with the *learning to learn* hypothesis. In fact, both gaming groups were exposed to the same subtasks and abilities required in FPS action videogame, such as flexibility, switching, and rapid response times (Colzato et al., 2010). A possibility is that a longer training period is necessary to highlight the eventual significant differences on cognitive effects between A-CS:GO and D-CS:GO.

## Conclusion

Findings further support published evidences that videogame playing might impact cognitive functioning in a beneficial way. Moreover, we demonstrate how a personalized/adaptive FPS experience can significantly speed-up the learning curve of action videogame players, opening to possible future applications for video-gamers, as well as potential rehabilitative applications in clinical populations.

## Data Availability Statement

The raw data supporting the conclusions of this article will be made available by the authors upon request to the corresponding author, without undue reservation.

## Ethics Statement

The studies involving human participants were reviewed and approved by Brainsight – Dipartimento di scienze mediche chirurgiche e neuroscienze – UOC Neurologia, Neurofisiologia, Policlinico Santa Maria alle Scotte, Siena, Italy. The participants provided their written informed consent to participate in the study.

## Author Contributions

ES, CS, and FN conceptualized and designed the study protocol. FN, DM, GS, CS, and SF collected the data. FN and ES performed the statistical analysis. AR, GDL, and ES oversaw the study conduction. FN, AM, and ES edited the first draft. SR and AR supervised the study and manuscript. ES, SR, FN, and AR critically reviewed the manuscript for content and all authors approved the final version for publication.

## Conflict of Interest

The authors declare that the research was conducted in the absence of any commercial or financial relationships that could be construed as a potential conflict of interest.

## Footnotes

1 www.eslgaming.com

2 www.pstnet.com/eprime
