# Supplementary material for: Personalized Adaptive Training Improves Performance at a Professional First-Person Shooter Action Videogame
Source: Front Psychol. 2021 Jun 10;12:598410. doi: 10.3389/fpsyg.2021.598410 (PMC8224404; doi:10.3389/fpsyg.2021.598410)
Supplement: Supplementary file 1 [file Data_Sheet_1.docx]

**Personalized Adaptive Training Improves Performance at a Professional First-Person Shooter Action Videogame**

Francesco Neri^1^, MS, Carmelo Smeralda^1^, MD, Davide Momi^1^, MS, Arianna Menardi^1^, MS, Giulia Sprugnoli^1^, MD, Simone Rossi^1,2,3^, MD, PhD, Alessandro Rossi^1,4^, MD, PhD, Giorgio Di Lorenzo^5,6^, MD, PhD, Emiliano Santarnecchi^1,7^, PhD

^1^ Siena Brain Investigation & Neuromodulation Lab, Department of Medicine, Surgery and Neuroscience, Neurology and Clinical Neurophysiology Section, University of Siena, Italy

^2^ Siena Robotics and Systems Lab (SIRS-Lab), Engineering and Mathematics Department, University of Siena, Italy

^3^ Human Physiology Section, Department of Medicine, Surgery and Neuroscience, University of Siena, Siena, Italy

^4^ Department of Medicine, Surgery and Neuroscience, University of Siena, Siena, Italy

^5^ Psychiatry and Clinical Psychology Unit, Department of Neurosciences, Fondazione Policlinico “Tor Vergata”, Rome, Italy

^6^ Laboratory of Psychophysiology, Psychiatric Chair, Department of Systems Medicine, University of Rome “Tor Vergata”, Rome, Italy

^7^ Berenson-Allen Center for Non-Invasive Brain Stimulation, Beth Israel Deaconess Medical Center, Harvard Medical School, Boston, MA, USA

**Supplementary information**

**Material and Methods**

**Cognitive Assessment Battery**

**Material and Methods**

**Cognitive Assessment Battery**

All cognitive tasks were presented using E-Prime 2.0 Professional (Psychology Software Tools) on a 19-inches screen located 80 cm away from the subject. A battery of cognitive tasks was administered for both experimental groups during a dedicated visit before (T0) and immediately after (T1) the experiment. A time window of 24 hours was left in between the end of the last game session and the cognitive re-test to avoid that the higher transient arousal following action game playing could altered tasks’ performance (Bavelier et al., 2012). Furthermore, the cognitive battery was administered after 3 months (T2) to verify possible long-lasting effects. Two subjects were not available for the T2 cognitive follow-up assessment (1 female and 1 male of the A-CS:GO group). Then, 19 subjects (A-CS:GO group 9 subjects, 3 females/6 males; 23.8 ± 2.5 years) and CS:GO (10 subjects, 2 females/8 males; 24.1 ± 2.3 years) were included in the statistical analysis conducted on the cognitive tasks performances.

The tests administered at all the three time points included widely validated tasks used previous studies: Visual Search, Global Local Features Task, Attentional Blink, Mental Rotation, Flanker Task, Serial Reaction Time Task and the Useful Field of View. Reaction time and accuracy were considered for the statistical analysis. Details on each task are reported below.

***Visual Search.*** In cognitive psychology, visual search relies on C of use in monitoring the visual environment to detect specific targets among a variable number of distractors (Treisman & Gelade, 1980). In the present study, a four-quadrant structure was presented with randomly placed "T" letters (i.e. distractors) on a black background screen. Participants were asked to identify, as quickly and as accurately as possible, the quadrant where an “L” letter (i.e. target) appeared by pressing the key on the pc keyboard corresponding to the quadrant where the L appeared. Overall, the “L” letter occurred 18 times for each quadrant, for a total amount of 72 trials. A fixation cross lasting 2000ms was shown at the centre of the screen, indicating the beginning of each trial. Participants were asked to respond within a maximum of 5 seconds, after which the next trial appeared.

***Global Local Features Task.*** Compound letter stimuli were used in the Global Local task to evaluate inhibition, attentional and switching skills. At the beginning of each trial, a written word (either “Local” or “Global) appeared at the centre of the screen to indicate whether if participants had to allocate their attention to the global or local features of the presented stimulus (e.g. a big H letter made of small S letters). If the written word was “Local”, participants were required to focus on the small letters which compounded the bigger letter, whereas if the word “Global” appeared on the screen, they had to pay attention to the big letter. Small and big “O”, “S” and “H” letters were used as stimuli, however the correct response could be either “S” or “H”, whilst “O” was employed as a distractor.

Different degrees of difficulty could therefore be set depending on whether participants experienced a Consistent, Neutral or Conflicting condition. The visual stimulus was considered Consistent when both the small letter and big letter matched. When either the big or the small letter were made up by the distractor (“O” letter), the visual stimulus was considered as Neutral. The hardest level was instead achieved in the Conflicting modality, when both the possible responses (“S” or “H”) were included in the visual stimulus. Participants were instructed to press as quickly and as accurately as possible the “S” key with their left index finger or the “H” key with their right index finger to solve the task. Each trial lasted up to a maximum of 8s, following which the next stimulus appeared. A total amount of 72 trials was administrated and inverse efficacies were calculated.

***Attentional Blink***. Attentional Blink is a phenomenon reflecting the difficulty of the visual system to allocate attentional resources on two consecutive stimuli when they are presented too close in time. It is usually investigated by means of streams of visual stimuli, where subjects typically fail to detect the second stimulus when it is presented within a range of 200-500 ms after the first one (Raymond et al., 1992; Chun & Potter, 1995; Green & Bavelier, 2003). In the present study, subjects carried out a training block consisting of 9 consecutive trials. At the beginning of each trial, a fixation asterisk appeared on the screen for 180 ms followed by a rapid stream of black letters. A first target consisting of a white letter (B, G or S) was randomly presented once in each trial and was immediately followed by a second target, a letter X, appearing in the 50% of the trials. Participants were asked to identify the identity of the first target letter by pressing the corresponding letter on the pc keyboard, and to subsequently indicate whether if the letter X appeared or not. No time limit was imposed to the participants in providing a response. The ability of the subject to detect or not the presence of the X-letter represented our variable of interest, as it was indicative of the cost of the attentional switching between the first and the second target.

***Mental Rotation.*** This task was used to evaluate subject’s ability to mentally rotate two-dimensional objects (Shepard & Metzler, 1971). A number “2” or a letter “R” appeared on the screen with randomized rotations either mirrored or reversed. Participants had to recognize whether the stimulus was rotated or mirrored and subsequently indicate it by pressing two different letters on the keyboard. A feedback was provided after the response was given. Each subject performed a training block of 24 trials.

***Flanker Task.*** The Flanker Task allows to evaluate inhibition skills and the ability of the subject to suppress responses that are inappropriate for a particular context (Eriksen, 1995). We used an adaptive version of the task (Green & Bavelier, 2006), in which six rings are presented briefly (16 ms) in the centre of the screen, with different geometrical shapes appearing either inside or outside the rings. A fixation cross lasting 1000ms informed participants of the beginning of each trial, whereas another fixation cross (lasting 500 ms) was presented at the end of the trial, following which subjects had to answer to the question “Was there either a square or a triangle inside one of the rings?” (i.e. forced choice). The distractors could be other geometrical shapes presented in the six rings (incompatible distractors), or squares and triangles presented outside the six rings (compatible distractors). Four levels of difficulty were presented depending on the type (compatible/incompatible) and the number (1-6) of distractors.

At the beginning, participants got acquainted with the task in performing an initial training block composed of 5 trials with feedbacks. Once at ease with the task, each subject performed 360 trials.

Considered the length of the protocol and to avoid tiredness, participants could stop the task and rest every time they needed by pressing the spacebar. Once ready, they could re-enter the task by pressing Enter on the keyboard.

***Serial Reaction Time Task.*** The Serial Reaction Time Task (SRTT) (Robertson, 2007) is considered a measure of visuo-motor learning. In this task, a visual cue is presented at four different positions, configured along the horizontal axis. Participants are asked to indicate the position of the visual cue on the screen by pressing the corresponding keyboard button. Blank intervals of 1000 ms of duration separate the presentation of a visual cue from the next.

In our study, participants performed a training block consisting of 5 trials. Once at ease with the task, they were presented with 3 blocks of 120 trials each, for a total amount of 360 trials. Subjects were allowed to take a break at the end of each block, and could continue the task when ready by pressing the spacebar. The position of the visual cue in each trial could either follow a repeating sequence or be random, such as that at the end of the task, participants fulfilled a questionnaire asking whether they have recognized a repeated sequence or not. Subjects were completely unaware about the questionnaire before the task.

***Useful Field of View.*** The Useful Field Of View (UFOV) refers to the visual field over which information can be caught with a rapid glimpse without eye or head movements (Ball et al., 1993; Edwards et al., 2006). Specifically, it measures the ability of the attentional system to locate a target among distractors. In the present study, an adapted UFOV task was implemented (Green & Bavelier, 2003; Feng et al., 2007; Spence et al., 2009), to evaluate whether if intensive action videogaming could modulate the allocation of attentional and spatial distribution resources.

A fixation square (0,66 x 0,66cm) lasting 600 ms indicated the beginning of the trial, following which a grid of 24 squares (distractors) and 1 circle (target) was flashed at the centre of the computer screen. A mask stimulus was shown for 600ms, until when a second grid (response cue) appeared. When the response cue was presented, participants had to determine as quickly and as accurately as possible in which of the eight possible directions the circle had appeared within the spoked structure. No feedback or time limit was given. Each square was uniquely localized at an eccentricity of 2,3 cm - 5,3cm or 8,3cm in one of eight equally spaced directions. The target stimulus could also be presented at different eccentricities, assessing spatial distribution of visual attention. Each trial was repeated 5 times, for a total amount of 120 trials. Before former testing, participants carried out a training block made up of 5 trials with feedbacks.

**References**

Ball, K., Owsley, C., Sloane, M. E., Roenker, D. L., & Bruni, J. R. (1993). Visual attention problems as a predictor of vehicle crashes in older drivers. *Investigative Ophthalmology & Visual Science*, *34*(11), 3110–3123.

Bavelier, D., Green, C. S., Pouget, A., & Schrater, P. (2012). Brain Plasticity Through the Life Span: Learning to Learn and Action Video Games. *Annual Review of Neuroscience*, *35*(1), 391–416. https://doi.org/10.1146/annurev-neuro-060909-152832

Chun, M. M., & Potter, M. C. (1995). A two-stage model for multiple target detection in rapid serial visual presentation. *Journal of Experimental Psychology. Human Perception and Performance*, *21*(1), 109–127.

Edwards, J. D., Ross, L. A., Wadley, V. G., Clay, O. J., Crowe, M., Roenker, D. L., & Ball, K. K. (2006). The useful field of view test: Normative data for older adults. *Archives of Clinical Neuropsychology*, *21*(4), 275–286. https://doi.org/10.1016/j.acn.2006.03.001

Eriksen, C. W. (1995). The flankers task and response competition: A useful tool for investigating a variety of cognitive problems. *Visual Cognition*, *2*(2–3), 101–118. https://doi.org/10.1080/13506289508401726

Feng, J., Spence, I., & Pratt, J. (2007). Playing an Action Video Game Reduces Gender Differences in Spatial Cognition. *Psychological Science*, *18*(10), 850–855.

Green, C. S., & Bavelier, D. (2003). Action video game modifies visual selective attention. *Nature*, *423*(6939), 534–537. https://doi.org/10.1038/nature01647

Green, C. S., & Bavelier, D. (2006). Effect of action video games on the spatial distribution of visuospatial attention. *Journal of Experimental Psychology: Human Perception and Performance*, *32*(6), 1465–1478. https://doi.org/10.1037/0096-1523.32.6.1465

Raymond, J. E., Shapiro, K. L., & Arnell, K. M. (1992). Temporary suppression of visual processing in an RSVP task: An attentional blink? *Journal of Experimental Psychology. Human Perception and Performance*, *18*(3), 849–860.

Robertson, E. M. (2007). The Serial Reaction Time Task: Implicit Motor Skill Learning? *Journal of Neuroscience*, *27*(38), 10073–10075. https://doi.org/10.1523/JNEUROSCI.2747-07.2007

Shepard, R. N., & Metzler, J. (1971). Mental rotation of three-dimensional objects. *Science (New York, N.Y.)*, *171*(3972), 701–703.

Spence, I., Yu, J. J., Feng, J., & Marshman, J. (2009). Women match men when learning a spatial skill. *Journal of Experimental Psychology: Learning, Memory, and Cognition*, *35*(4), 1097–1103. https://doi.org/10.1037/a0015641

Treisman, A. M., & Gelade, G. (1980). A feature-integration theory of attention. *Cognitive Psychology*, *12*(1), 97–136. https://doi.org/10.1016/0010-0285(80)90005-5
